# Causal ECGNet: leveraging causal inference for robust ECG classification in cardiac disorders

**DOI:** 10.3389/fphys.2025.1543417

**Published:** 2025-05-19

**Authors:** Mei Wang, Cong You, Wei Zhang, Zibo Xu, Qi Liang, Qiang Li

**Affiliations:** ^1^ Department of Dermatology, Tianjin First Central Hospital, Tianjin, China; ^2^ Department of Dermatology and Venereology, The First Affiliated Hospital of Gannan Medical University, Ganzhou, China; ^3^ Research Department 3, Nanjing Research Institute of Electronic Engineering, Nanjing, China; ^4^ School of Microelectronics, Tianjin University, Tianjin, China; ^5^ Tianjin Navigation Instruments Research Institute, Tianjin, China

**Keywords:** ECG classification, attention, time domain features, causal reasoning, backdoor adjustment, infectious disease diagnosis, cardiac signal variability

## Abstract

Electrocardiogram (ECG) is a graphical representation of the electrical activity of the heart and plays a crucial role in diagnosing heart disease and assessing cardiac function. In the context of infectious diseases, ECG classification is particularly critical, as many infections, such as viral myocarditis and sepsis, can cause significant cardiac complications. Early detection of infection-induced cardiac abnormalities through ECG can provide timely intervention and improve patient outcomes. However, current ECG processing methods often overlook the impact of confounding factors caused by statistical associations, which can compromise classification accuracy, especially in infection-related cardiac conditions. To address this, we propose an innovative approach to causal reasoning based on attention mechanisms. By employing backdoor adjustment for each cardiac lead, our method effectively eliminates confounding factors and models the true causal relationship between ECG patterns and underlying cardiac abnormalities caused by infectious diseases. Furthermore, our approach integrates the concept of entropy with causal inference to enhance ECG classification. By quantifying the information content and variability in ECG signals, we can better identify patterns and anomalies associated with infection-induced cardiac conditions. Experimental results demonstrate that our method achieves significant improvements in classification accuracy and robustness across four benchmark ECG datasets, outperforming existing methods. This work provides a novel perspective on the interplay between infection and cardiac function, offering valuable insights into the detection and understanding of infection-related cardiac complications.

## 1 Introduction

Cardiovascular disease (CVD) remains the leading cause of mortality worldwide, accounting for approximately 20.5 million deaths in 2021, which represents about one-third of all global deaths (Federation, 2023). While high-income countries have made significant strides in cardiovascular health, this progress remains uneven, particularly in low- and middle-income countries, where over 75% of CVD-related deaths occur. These regions often face limited access to specialized cardiology services, further exacerbating the challenge of timely diagnosis and intervention. Moreover, infectious diseases such as COVID-19, HIV, and other viral or bacterial infections have been shown to exacerbate or directly contribute to cardiovascular complications, including myocarditis and sepsis-induced cardiac dysfunction. This dual burden of CVD and infectious diseases underscores the urgent need for accurate, automated tools to interpret electrocardiograms (ECG) and support diagnosis in resource-limited settings. In recent years, machine learning (ML) has played a pivotal role in advancing ECG signal classification (Jekova et al., 2008; Ince et al., 2009), offering new possibilities for understanding the interplay between infectious diseases and cardiovascular health. The ECG, as a critical test in the medical field, reveals the heart’s electrical activity from multiple dimensions. Machine learning algorithms, through their ability to analyze large datasets, identify complex patterns, and classify a wide range of cardiac conditions, are transforming cardiovascular care (Andreao et al., 2006; Martis et al., 2013; Karimifard et al., 2006). For regions heavily impacted by infectious diseases, these algorithms hold promise for detecting infection-induced cardiac abnormalities, such as myocarditis caused by viral infections. Additionally, real-time monitoring systems incorporating unsupervised learning and anomaly detection methods, such as auto-encoders, provide continuous analysis of ECG data, enabling timely warnings for healthcare providers. These technologies not only support early detection of cardiovascular issues but also offer valuable insights into the cardiac complications associated with infectious diseases, facilitating better clinical outcomes and resource allocation.

**FIGURE 1:**
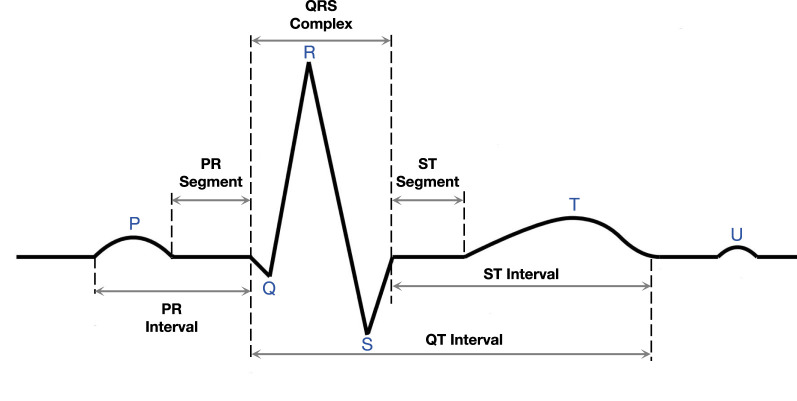
Part of an electrocardiogram.

In the traditional diagnosis, a healthcare professional performs a detailed analysis of the ECG, focusing on the shape, duration, and temporal characteristics of electrical waveforms, including P waves, QRS wave groups, and T waves, which are illustrated in Figure 1. Through careful observation and interpretation of these characteristics, medical personnel can deeply understand the heart condition of patients, and then provide an accurate basis for the diagnosis and treatment of cardiovascular problems. ECG signals play a crucial role in diagnosing various cardiac disorders, including those induced by infectious diseases such as viral myocarditis and sepsis-related myocardial injury (Siontis et al., 2021). In these conditions, the infectious agents or systemic inflammatory responses can directly or indirectly affect the heart, leading to characteristic changes in ECG waveforms. For instance, viral myocarditis may manifest as ST-segment elevation, T-wave inversion, or arrhythmias, while sepsis-related myocardial injury often results in prolonged QT intervals or reduced heart rate variability. The detection and classification of these ECG patterns are essential for early diagnosis and timely intervention in infection-related cardiac disorders.

In the field of computer vision, the judgment of the model is often disturbed by a variety of confounding factors, which makes accurate image analysis more complex and challenging (Nie et al., 2023a). Confounding factors can include illumination change, shadow, noise, occlusion, and other environmental or sensor-related artifacts. These factors not only affect the quality of the image but also confuse the model’s correct understanding of the image content. Similarly, in the context of ECG signal analysis, confounding factors manifest in different yet equally impactful ways. For instance, variations in time-scale features, inter-lead differences, basic physiological characteristics of patients, and prior information on disease types can all act as confounders (Nie et al., 2023b). Time-scale features, such as short-term fluctuations and long-term trends, are directly observable and extracted using multi-scale convolutional kernels. Inter-lead differences, particularly in multi-lead ECG signals like 12-lead ECG, reflect the varying characteristics of signals from different leads, which can significantly influence classification results. Basic physiological features, such as heart rate, QRS complex morphology, and T-wave morphology, are inherent components of ECG signals and may simultaneously affect the signals and the classification outcomes. Additionally, prior information on disease types, derived from the labels in the training data, allows us to construct a confounding dictionary by computing the average features for each class, representing the typical confounding characteristics of different classes (Liu et al., 2024; Nie et al., 2024).

It is worth emphasizing that the existence of confounding factors not only makes the judgment of the model fuzzy and uncertain, but these factors themselves may interweave and influence each other to form a more complex visual environment. For example, in ECG signals, patient-specific characteristics such as age, gender, or body composition may interact with recording conditions like electrode quality or environmental noise to create compounded distortions that are difficult to disentangle. However, the discovery and removal of confounding factors in ECG signals is also a challenging task (Sajjan, 2012), as they may take on subtle and imperceptible forms in the signal that are difficult to capture by simple rules or algorithms. If the model deduces based on these confounding features, it is easy to make the wrong judgment because the model does not consider the true causal relationship between the features and the label.

These confounders are considered visible because they can be directly extracted from the ECG signals or data labels without requiring additional assumptions or unobservable variables. Time-scale features and inter-lead differences are extracted using signal processing techniques such as convolutional operations, while basic physiological features are obtained through standard ECG analysis methods. Prior information on disease types is derived directly from the labels in the training data, making it known and observable (Liu et al., 2024). Our approach assumes that these confounders are observable and can be effectively addressed through the construction of a confounding dictionary and causal interventions, such as backdoor adjustment. This assumption is based on the fact that these confounders are directly extracted from the data, eliminating the need for additional unobservable variables, and by constructing a causal graph, we explicitly model the relationships between these confounders, ECG signals, and classification outcomes (Nie et al., 2024; Ribeiro et al., 2020).

Besides, if we ignore time-scale features and only rely on local information to apply the traditional method to ECG signal classification, it will lead to the failure to fully understand the rich information contained in ECG signals (Bender et al., 2023). A single-scale analysis method may lead to an oversimplification of the characteristics of the ECG signal, thus affecting the final classification accuracy. Therefore, in order to interpret ECG signals more comprehensively, we introduce a time-domain features embedding module. By learning and utilizing the features of different time scales, we can better capture the dynamic features of the signal and improve the performance and generalization ability of the classification algorithm. When the ECG signal is used as input, the model is easily disturbed by confounding factors (Miao et al., 2023; Zhan et al., 2023). Confounding factors, such as individual differences, the complexity of the disease, and other physiological disturbances, have a non-negligible impact on the accurate classification of ECG signals. Traditional approaches often fail to fully account for these confounding factors, resulting in models that perform poorly in the face of complexities. Especially in complex clinical settings, the presence of these confounding factors may mask the true causal relationship and reduce the reliability and generalization performance of the classification model.

In order to overcome this challenge, a causal reasoning method was introduced in this study, aiming at deeply mining the correlation in ECG signals, clarifying the causal chain, and effectively eliminating the interference of confounding factors, thus improving the adaptability of the classification model to complex situations (Ghongade and Ghatol, 2007; Nagal and Sharma, 2014). Specifically, we propose a classification network for ECG signals based on causal reasoning. First, in the feature mapping part of the whole network, we use convolution kernels of different sizes. Given the diversity of ECG signals on time scales, the characteristics of different time scales are essential for a comprehensive understanding of the signals. At the same time, the module considers various perspectives of the ECG signal to capture more abundant feature information. This allows the network to consider time dynamics more comprehensively when analyzing ECGs, thus improving the accuracy of the classification algorithm. Second, the causal reasoning module is the de-confounding part of our proposed network. Confounders are often a major obstacle to model performance in ECG signal classification tasks. In the causal reasoning module, we adopt the backdoor adjustment method to eliminate the influence of confounding factors by constructing a causal graph and cutting off the false causal path, so as to realize the identification of a real causal correlation. The causal module ensures that the model is more reliable and stable in the classification process and avoids misjudgments caused by confounding factors.

Given the preceding discussion, the key achievements of the study can be outlined as follows:

• We mitigate the interference of confounding factors in ECG signals by introducing backdoor adjustment, allowing for the preservation of true causal features associated with infection-induced cardiac abnormalities. This approach enhances network classification performance and provides a robust framework for accurately identifying cardiac conditions influenced by infectious diseases.
• We leverage the time-domain diversity of ECG signals to enable the causal reasoning module to effectively address confounding factors arising from temporal variations in ECG data. By eliminating the adverse impact of these factors, our method improves classification accuracy and robustness, particularly in detecting infection-related cardiac anomalies.
• Results of extensive experiments on four multi-label ECG datasets demonstrate that our method surpasses the existing advanced networks across several classification tasks.

## 2 Related work

### 2.1 Causal learning for medical images

In the past few years, many research has been devoted to the application of causal learning methods to medical image processing, covering medical image classification, image segmentation, and medical question and answering tasks.

Nie et al. (Nie et al., 2023a; Nie et al., 2023b; Liu et al., 2024) used a variety of causal learning methods in medical image classification. They succeeded in removing visible confounding factors by using backdoor adjustment methods. In addition, they cleverly introduced instrumental variables, extracting information from chest imaging and electronic health record sheets to effectively eliminate invisible confounding factors. Miao et al. (2023) proposed a method of medical image segmentation based on causal graphs, which provides a more interpretable approach for semi-supervised learning of medical image segmentation. In their research, they emphasize the importance of algorithmic independence based on the principle of cause-effect graphs, and design an innovative statistical quantification method to approximate uncomputable algorithmic independence. Zhan et al. (2023) propose an innovative debiased medical visual question-answering (MedVQA) model, which cleverly incorporates counterfactual data during the training phase and directly subtracts the causal effects of linguistic priors, thus successfully migrating linguistic biases in the final MedVQA. This approach not only focuses on the influence of linguistic priors, but also powerfully mitigates bias in training by introducing counterfactual data. However, while existing research has made significant progress in tasks such as medical image classification, segmentation, and intelligent question and answer, the unique nature and data complexity of ECGs make causal reasoning in this field even more complicated.

### 2.2 Machine learning for ECG signal classification

Jekova et al. (2008) explored morphological features, examining characteristics like the amplitude and width of QRS complex waves. On the other hand, statistical features are obtained using techniques such as wavelet transform or hidden Markov chain (Ince et al., 2009; Andreao et al., 2006). Nagal et al. (Martis et al., 2013) proposed some mathematically based analytical methods to deal with high-dimensional features. In feature classification, algorithms like multilayer perceptron (Karimifard et al., 2006), K-nearest neighbors (Ghongade and Ghatol, 2007), and support vector machines (Nagal and Sharma, 2014) are frequently utilized. Martis et al. (Shimpi et al., 2017) proposed principal component analysis to reduce ECG signal dimensionality, followed by using a visual word bag method, involving feature block extraction, codebook construction, and pooling strategies for generating the final features to the SVM for classification. Lassoued and Ketata, (2018) proposed discrete wavelet transform coefficients for feature extraction, with a fully connected layer serving as the classifier. However, machine learning methods are hindered by their reliance on meticulous data preprocessing, making it challenging to achieve optimal performance.

Wang et al. (2021) introduced a method that integrates a 33-layer CNN with a attention module. This CNN is adept at extracting spatial and channel features, while the non-local attention mechanism effectively captures long-range dependencies across these dimensions. Chen et al. (2020) proposed an effective ECG classification network comprising five CNN blocks, bidirectional GRU with an attention mechanism. Yang et al. (2023) believed that by extracting the features of ECG signals from multiple views, higher quality features can be obtained compared to a single view. Finally, multiple features are fused through a multi-view fusion module to get the final feature. Zhang et al. (2023) proposed an efficient method to learn multi-scale features by using a two-branch structure. Han et al. (2023) proposed a fusion module for ECG signal classification based on multi-modal and attention mechanisms, aiming at preprocessing methods that are easy to lead to the loss of key information. In addition, in order to avoid extracting only a single feature which makes it difficult for the model to learn the complete ECG information, they use both the time domain information and the visual domain information of the ECG through a multi-modal method.

## 3 Methods

### 3.1 Method overview

In this section, we will describe our ECG classification method in detail. Unlike traditional medical image classification tasks, an ECG is not an image, but a signal pattern. The ECG signal consists of multiple waveforms, including the P wave, QRS wave group, and T wave (see Figure 1). The classification basis of the model is to classify diseases according to the changes and anomalies of these waveforms. The whole classification process includes signal preprocessing, feature extraction, feature decoupling, and disease classification. Unlike traditional medical images, an ECG consists of sub-images from multiple perspectives. This feature provides more abundant information for classification tasks, but also inevitably introduces the influence of confounding factors.

**FIGURE 2:**
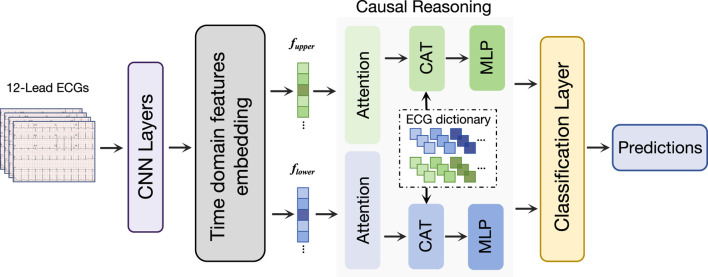
Overview of our proposed network. Our network first processes the ECG signals through the convolutional layer, and then obtains the time-scale features through the time domain features embedding module. We designed a causal reasoning module for ECG classification to perform feature decoupling. Finally, a classification layer is used to make classification predictions.

Therefore, as shown in Figure 2, we propose a causal inference classification framework based on attention mechanisms. First, we use convolutional layers to extract preliminary features, and time-domain features embedding module consisting of convolution kernel of different sizes are used to further extract time-domain features of ECG signals. The separated temporal features are then fed into their respective causal reasoning modules, utilizing novel attention mechanisms to capture true causal relationships. After causal learning, the characteristics of causal information are selectively retained according to causal relationships. Finally, the classification task is completed by the classification layer, and the result is predicted according to the causal characteristics.

### 3.2 Convolution layers

We use the entire ECG as input, so the input consists of signals from multiple angles, represented as $X^{m}\in R^{n\times L}$ , where m∈{1,2,…,6} , n is the number of leads, and L is the signal length. Here, $X^{m}$ denotes the m -th view signal, which corresponds to a specific spatial perspective of the heart. Specifically, the 12-lead ECG signal is divided into six view signals, each capturing a unique spatial angle or perspective of the heart. Each $X^{m}$ is a matrix $\in R^{n\times L}$ , with n=2 (since the 12-lead ECG is divided into six pairs of leads) and L representing the length of the signal in time steps. Each $X^{m}$ is then entered into the network corresponding to the m -th view.

The m -th view can be expressed as follows:

$$f^{m}=Cv\left(X^{m}\right),$$ (1)

where Cv represents a convolutional layer, and $f^{m}$ indicates the preliminary ECG features extracted from the m -th view. In Equation 1 each view network shares the same structure but processes the signals from different spatial perspectives, enabling the model to capture comprehensive information from the ECG.

In an ECG, the convolution kernel is responsible for detecting specific waveform features, such as P waves, QRS wave groups, and T waves. This convolution operation is carried out on the whole signal, and through the mechanism of weight sharing, the convolution kernel learns and recognizes the same local features at different locations.

### 3.3 Time domain features embedding

We emphasize that in order to extract time information from the ECG, we have adopted an efficient method of using only a few convolution kernel of different sizes without the need to introduce a timing network. Various scale features in ECG reflect the information of different time scales. Oversimplifying the ECG to a single feature block will lead to ignoring the correlation between the features in a specific time frame. Therefore, we introduce the time domain features embedding module, which can be viewed as a time-scale adaptive feature extractor.

The time domain features embedding module obtains more comprehensive time domain features by using convolution kernels of different sizes. Smaller convolution kernel are able to capture short-term signal fluctuations sensitively, while larger convolution kernel are better able to capture long-term signal trends. This feature extraction method helps the network to better understand the complex time domain structure of the ECG signal and improve the sensitivity of the heart condition. It is worth noting that the module can not only avoid the information loss caused by a single feature extraction method, but also enable the network to consider and analyze the signal features at different times more comprehensively. The time-scale embedding process can be expressed as:

$$f_{\mathrm{upper}}=T_{1}\left(f^{m}\right),f_{\mathrm{lower}}=T_{2}\left(f^{m}\right),$$ (2)

where T indicates the time feature mapping module. In Equation 2 we integrate this module into our model to ensure a reliable ECG classification network capable of capturing features at different time scales (Gao et al., 2019; Nie et al., 2024).

Specifically, we use two one-dimensional cores of different sizes, namely, 1×5 and 1×50 , to achieve the organic combination of global comprehensive feature information and local specific feature details. The goal is to more accurately capture the signal characteristics of local areas and improve the sensitivity of the network to microscopic details. This layered design allows the network to better adapt to signal changes over different time domains, not only helping to emphasize the detailed structure of signals in local regions, but also covering changes over different time scales more comprehensively. Through the time domain features embedding layer, we can capture the relevant information of heartbeat fragments in different time domains more comprehensively and thoroughly, and effectively improve the ability of the network to grasp cross-scale features.

Therefore, the role of this module in ECG classification is mainly reflected in its ability to effectively capture time domain diversity, improve the network’s perception of signal details and global features, and thus provide a more accurate and comprehensive feature representation for the final classification task. In this time domain features embedding module, we use different sizes of cores because the ECG data is one-dimensional, and using convolution cores of different sizes in the one-dimensional domain is better for collecting features on different time scales. For example, larger kernels provide a larger receptive field, enhancing their ability to capture features.

### 3.4 Causal learning

**FIGURE 3:**
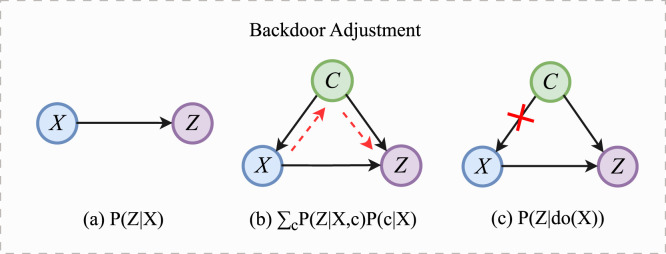
The process of backdoor adjustment. **(a)** X→Z denotes the true relationship between X and Z . **(b)** When confounder C exists, the true association between X and Z is disturbed. **(c)** Backdoor adjustment can eliminate interference by cutting off the backdoor path.

As shown in Figure 3a, when there are no confounders in ECG signals, P(Z|X) correctly denotes the true relationship between X and Z .

In Figure 3b, C is defined as the confounder because it is a common cause for both X and Z . Combined with the causal diagram, the influence of C on X can be expressed as C→X , and C→Z indicates that C also affects the prediction result. P(Z|X) can be represented according to Bayes’ rule:

$$P\left(Z|X\right)=\underset{c}{\sum }P\left(Z|X,c\right)P\left(c|X\right),$$ (3)

where the confounder C generally brings about the observed bias via P(c|X) .

Specifically, in the ECG classification task, we want to understand the influence of confounding factors C , such as specific waveform, time domain information, etc., on the result Z . However, in Equation 3 P(C|X) is based on the statistical association between X and C in the training set, rather than the causal association between the two. In the presence of dataset bias, the model tends to learn false associations between X and Z caused by C , that is, overuse the co-occurrence between visual context and class labels to learn false representations.

To eliminate the influence of confounding factors, it is crucial to close the backdoor path. For the backdoor path X←C→Z in Figure 3, we introduce causal intervention P(Z|do(X)) to block. The do calculus do () cuts off the backdoor path C→X . Backdoor adjustments can be expressed as:

$$P\left(Z|do\left(X\right)\right)=\underset{c\in C}{\sum }P\left(Z|X,c\right)P\left(c\right),$$ (4)

where P(c) represents the prior probability of c . Since c is visible, concatenating c with X can be used to eliminate false associations. In this way, the model can learn the true causality from X to Z to make correct predictions. Equation 4 signifies that to estimate the causal effect of X on Z , we sum over all possible values of the confounder C , weighting the conditional probabilities P(Z|X,C=c) by the marginal probabilities P(C=c) . This process adjusts for the influence of C , ensuring that the relationship between X and Z is not confounded.

After obtaining $P\left(Z|do\left(X\right)\right)=\sum_{c\in C}P\left(Z|X,c\right)P\left(c\right)$ , which is crucial for causal intervention, the next step is to integrate this causal reasoning into the classification process to eliminate confounding effects. To construct the confounder dictionary, we compute average feature vectors for each class based on features extracted by our time-domain feature embedding module. It is important to note that the feature vectors used for averaging are not raw signal representations, but high-level embeddings that incorporate multi-scale temporal information. Specifically, after passing through convolutional layers and the time-domain features embedding module—which captures both short-term and long-term temporal dependencies using kernels of different sizes—each sample is mapped to a feature vector that summarizes critical waveform characteristics, such as QRS complex morphology, ST-segment shifts, and T-wave patterns. For each class c∈C , we extract all corresponding feature vectors $f_{i}^{\left(c\right)}$ and compute the average vector: $f_{d}=\frac{1}{N_{c}}\sum_{i=1}^{N_{c}}f_{i}^{\left(c\right)}$ , where $N_{c}$ is the number of samples in class c . These average vectors are then stored in the confounder dictionary $f_{d}^{\left(c\right)}$ . Rather than eliminating variability, this approach captures the typical confounding patterns across a class while preserving individual differences through the feature extraction pipeline. By using averaged high-level representations—instead of raw or shallow features—we ensure that critical diagnostic features and temporal dynamics are retained during causal intervention, enhancing the robustness and generalization ability of the model without sacrificing sensitivity to key ECG variations. By leveraging these representations, the model can explicitly account for and mitigate the influence of such confounders during causal intervention. This approach provides a practical and efficient way to approximate latent confounders without requiring explicit annotations or additional data, enabling the model to dynamically access and adjust for class-specific confounding patterns, thereby improving its robustness and generalizability.

To estimate the probability distribution of a function f(x) , we calculate its expected value as follows:

$$E_{x}\left[f\left(x\right)\right]=\underset{x}{\sum }f\left(x\right)P\left(x\right),$$ (5)

where x represents the input variable, f(x) is a function of x , and P(x) denotes the probability distribution of x . Next, we define the Weighted Geometric Mean (WGM) of a function f(x) with respect to P(x) in Equation 5:

$$WGM\left(f\left(x\right)\right)=\prod_{x}f{\left(x\right)}^{P\left(x\right)}=\prod_{x}\exp\,{\left[g\left(x\right)\right]}^{P\left(x\right)}=\exp\left[\sum_{x}g\left(x\right)P\left(x\right)\right]=\exp\left[E_{x}\,\left[g\left(x\right)\right]\right]\approx E_{x}\,\left[f\left(x\right)\right],$$ (6)

where f(x)=exp[g(x)] and g(x) is a function of x . In Equation 6 the WGM provides a geometric average of f(x) weighted by P(x) . Building on this, the Normalized Weighted Geometric Mean (NWGM) approximation is defined as:

$$NWGM\left(f\left(x\right)\right)=\frac{\prod_{x}\exp{\left(g\left(x\right)\right)}^{P\left(x\right)}}{\sum_{j}\prod_{x}\exp{\left(g\left(x\right)\right)}^{P\left(x\right)}}=\frac{\exp\left(E_{x}\left[g\left(x\right)\right]\right)}{\sum_{j}\exp\left(E_{x}\left[g\left(x\right)\right]\right)}=\text{Softmax}\left(E_{x}\left[g\left(x\right)\right]\right).$$ (7)

The NWGM normalizes the WGM to ensure that the output is a valid probability distribution, suitable for use in classification tasks.

In our proposed method, the conditional probability distribution P(Z|X) is used as the predictive function of the output Z given the input X . It is natural to parameterize P(Z|X) as a neural network with a Softmax layer, such that:

$$P\left(Z|X\right)=\text{Softmax}\left[g\left(X\right)\right]\propto \exp\left[g\left(X\right)\right],$$ (8)

where g(X) is a function of X implemented by the network.

By combining the backdoor adjustment formula Equation 4 with the NWGM approximation Equations 7 and 8, we derive the interventional probability distribution P(Z|do(X)) as follows:

$$P\left(Z|do\left(X\right)\right)=\underset{c\in C}{\sum }P\left(Z|X,c\right)P\left(c\right)=E_{c}\left[F\left(X,c\right)\right]=\text{Softmax}\left(g\left(X,E_{c}\left[c\right]\right)\right),$$ (9)

where C represents the set of confounders, c is a confounding variable, and $E_{c}\left[c\right]$ is the expected value of c with respect to its distribution P(c) . Equation 9 allows us to efficiently compute the interventional distribution by leveraging the NWGM approximation, ensuring that the model’s predictions are robust to confounding biases.

During the classification process, we combine the obtained causal intervention probability P(Z|do(X)) with the confounding dictionary to eliminate confounding effects. Specifically, we compute the causal intervention probability P(Z|do(X)) for each sample feature X with respect to the target variable Z . Then, we multiply this probability by the corresponding confounding features, i.e., P(Z|X,c)P(c) multiplied by the confounding features for the corresponding class from the confounding dictionary. This process effectively removes the influence of confounding factors, enhancing the accuracy and stability of the classification. Through this approach, we not only achieve ECG classification based on causal reasoning but also effectively address the challenges posed by confounding factors, thereby providing robust support for improving classification performance.

### 3.5 Causal reasoning

In this study, we use an attention mechanism for causal reasoning (CR) to select true causal relationships. CR establishes connections among feature channels to reassess their significance and effectively capture spatial information within the feature space (Hou et al., 2021).

Attention mechanisms play a crucial role in enhancing network performance. Squeeze-and-Excitation (SE) attention (Hu et al., 2018), a widely used attention mechanism, is recognized for its efficiency with a minimal parameter count and outstanding performance. While channel attention focuses on constructing relationships between feature channels to reweight their importance, it often overlooks spatial information. However, spatial information is essential for generating spatially selective attention maps.

In our study, we applied an attention fusion module to the task of ECG classification, coupled with causal inference for backdoor adjustment. This step is particularly crucial when dealing with ECG data, as it often suffers from confounding factors such as patients’ physiological states and cardiac histories. We first input the features extracted from the ECG data as queries Q into the attention fusion module. These features contain vital information about cardiac signals but may be influenced by confounding factors. Subsequently, we input the confounder dictionary as keys K and values V into the module. The confounder dictionary contains the average features for each class, representing typical representations of various confounding factors. Within the attention fusion module, we dynamically fuse the features with the confounder dictionary using an attention mechanism to enhance feature representation and discrimination. Using the fused feature representation, we compute the backdoor adjustment P(Z|do(X)) , where Z represents the classification target and X represents the features. This process involves the core idea of causal inference, which infers causal relationships from observed data. In our study, leveraging the fused features obtained from the attention fusion module and combining them with causal inference methods, we effectively control potential confounding factors, thereby improving the accuracy and stability of ECG classification.

After time-scale embedding, the features are divided into upper features and lower features. These features pass through the causal reasoning module, in which true causal features are selected. The extraction process of causal features is as follows:

$$f_{\mathrm{cau1}}=MLP\left(F_{c}\left(f_{\mathrm{upper}}\right)\right),f_{\mathrm{cau2}}=MLP\left(F_{c}\left(f_{\mathrm{lower}}\right)\right),$$ (10)

In Equation 10, MLP denotes the multi-layer perceptron, $F_{c}$ denotes the transformer, $f_{\mathrm{cau}}$ denotes causal features.

**FIGURE 4:**
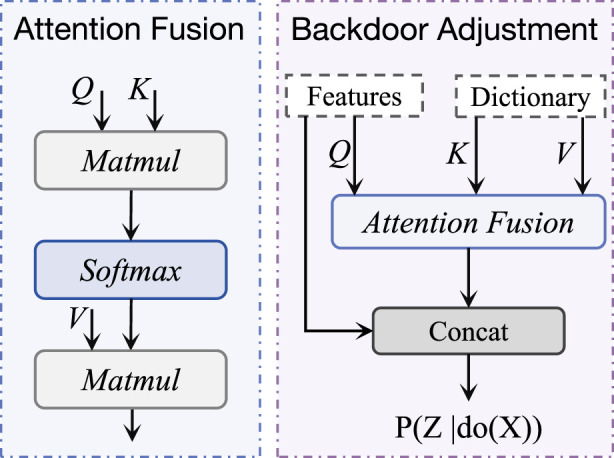
The structure of backdoor adjustment, which is based on the attention fusion module. It takes ECG features and confounding dictionaries as inputs to enable causal reasoning processes.

In terms of feature decoupling, we use an Attention Fusion (AF) module for causal reasoning. As shown in Figure 4, given the input, the attention operation in the AF module can be expressed as:

$$\begin{array}{cc} & \text{Attention}\left(Q,K,V\right)=\text{Softmax}\left(\frac{{QK}^{T}}{\sqrt{D}}\right)V,\\ & \;Q_{1}=f_{\mathrm{upper}},K_{1}=f_{d},V_{1}=f_{d},\\ & \;Q_{2}=f_{\mathrm{lower}},K_{2}=f_{d},V_{2}=f_{d}, \end{array}$$ (11)

In Equation 11, $f_{d}$ denotes features of the confounding dictionary. The AF module accepts the query (Q) , key (K) , and value (V) as input, first calculates the normalized attention distribution, which is then multiplied with the values to produce the final output.

The confounding dictionary is constructed by computing the average feature vector $f_{d}$ for each class, as described earlier. To transform these average features into keys and values for the AF module, we employ learnable linear projections. Specifically, for each class c , the average feature vector $f_{d}$ is mapped into a key $K_{c}$ and a value $V_{c}$ using the following transformations:

$$K_{c}=W_{k}f_{d}+b_{k},\;V_{c}=W_{v}f_{d}+b_{v},$$

where $W_{k}$ and $W_{v}$ are weight matrices, and $b_{k}$ and $b_{v}$ are bias terms. These parameters are optimized during training to ensure that the keys and values effectively capture the confounding patterns.

In the AF module, the keys $K_{1}$ and $K_{2}$ are constructed by aggregating the class-specific keys ${\left\{k_{c}\right\}}_{c=1}^{C}$ from the confounder dictionary. These keys are used to compute attention scores by measuring the similarity between the queries ( $Q_{1}$ and $Q_{2}$ ) and the keys. The attention scores are then normalized using the Softmax function and used to weight the corresponding values $V_{1}$ and $V_{2}$ , which are derived from the class-specific values ${\left\{v_{c}\right\}}_{c=1}^{C}$ . This process allows the module to dynamically retrieve and incorporate confounder-aware information into the feature representations, thereby mitigating the influence of spurious correlations.

By leveraging the confounder dictionary in this way, the AF module explicitly accounts for class-specific confounding factors, enhancing the model’s ability to learn robust and generalizable representations. This transformation of the confounder dictionary into keys and values is a critical component of our causal intervention strategy, enabling the model to disentangle causal relationships from confounding biases.

### 3.6 Training strategy

In causal reasoning, we want to estimate the true causal characteristics, so we use the cross entropy loss:

$$L_{\mathrm{ce1}}=-\frac{1}{\left|N\right|}\underset{n\in N}{\sum }y^{⊤}log\left(z_{\mathrm{cau1}}\right),L_{\mathrm{ce2}}=-\frac{1}{\left|N\right|}\underset{n\in N}{\sum }y^{⊤}log\left(z_{\mathrm{cau2}}\right),$$ (12)

In Equation 12, $f_{\mathrm{cau1}}$ and $f_{\mathrm{cau2}}$ are extracted from the causal reasoning module, corresponding to $f_{\mathrm{upper}}$ and $f_{\mathrm{lower}}$ respectively., n is a sample in the ECG training data N . In the training stage, the objective of our framework can be defined as the sum of the losses:

$$L_{\mathrm{total}}=\alpha_{1}L_{\mathrm{ce1}}+\alpha_{2}L_{\mathrm{ce2}},$$ (13)

In Equation 13, $L_{FC}$ is the feature correlation loss, $\alpha_{1}$ and $\alpha_{2}$ are the hyperparameters that control the trade-off between the primary task loss and the feature correlation loss.

## 4 Experiment

### 4.1 Datasets

The PTB-XL dataset (29), which is a recently introduced and comprehensive collection of 12-lead ECG dataset, consists of 21,837 clinical ECG recordings. Each recording spans a duration of 10 s and is sourced from a total of 18,885 individual patients. Table 1 provides an overview of the amount of ECG recordings in various tasks within this dataset. Table 1 summarizes the distribution of ECG recordings across various tasks in the PTB-XL dataset, including rhythm, form, super-diagnosis (super-diag.), sub-diagnosis (sub-diag.), diagnosis (diag.), and all. For each task, the table provides the number of classes, as well as the number of recordings in the training, validation, and test sets, along with the total count. The tasks are designed to capture different levels of ECG interpretation, ranging from broad rhythm classification to detailed diagnostic categorization. This table serves as a comprehensive reference for understanding the dataset’s structure and facilitates the development and evaluation of models across diverse ECG analysis tasks. The CPSC (Liu et al., 2018) comprises 6,877 12-lead ECG recordings, each spanning from 6 to 60 s. The HFHC (Anonymous, 2019) comprises 20,335 medical ECG samples, categorized into 34 classes, and each sample is equipped with 8 leads. The Chapman (Zheng et al., 2020), consisting of 12-lead ECG recordings from 10,646 patients, is utilized in this study.

**TABLE 1:** Amount of ECG recordings in the training set, validation set, and test set in various tasks on the PTB-XL dataset.

Task	Classes	Train	Val	Test	Total
rhythm	12	16854	2,109	2,103	21033
form	19	7202	904	882	8988
super-diag	5	17111	2,156	2,163	21430
sub-diag	23	17111	2,156	2,163	21430
diag	44	17111	2,156	2,163	21430
all	71	17441	2,193	2,203	21837

### 4.2 Implementation details

For each dataset, the samples are randomly divided following an 8:1:1 ratio into training, validation, and test sets. Approximately 80% of the data is used for training the model and capturing latent patterns within the ECG signals. Around 10% of the data is reserved for validation, which is utilized for model selection and hyperparameter tuning. The remaining 10% is set aside as an independent test set to assess the model’s generalization ability. For each dataset, we partition the data into 10 non-overlapping groups. Specifically, groups 1 to 8 are used as the training set to train the network and learn latent patterns from the data. Group 9 is employed as the validation set for model selection and hyperparameter tuning. Group 10 serves as an independent test set to evaluate the performance of the model on unseen data. All experiments are conducted with standardized training settings to ensure consistency and comparability across datasets. The optimizer used is Adam, with an initial learning rate of 0.001. The learning rate is scheduled to decay following a cosine annealing strategy during training. The batch size is set to 64 for all experiments. Cross-entropy loss is adopted as the loss function. The implementation is based on the PyTorch framework. All experiments are performed on a single NVIDIA RTX 3090 GPU with 24 GB memory. This standardized setup facilitates reproducibility and fair comparison across different datasets.

### 4.3 Experimental results

In the experiment of ECG multi-label classification task, we obtained the results in Table 2 by comparing the performance of our proposed method with other advanced methods on the PTB-XL dataset. In the table, we show the classification performance of various methods on different tasks (rhythm, form, super-diag., sub-diag., diag., all), using sensitivity (SEN) and area under the curve (AUC). For the rhythm task, our method achieves 91.68% on SEN and 97.02% on AUC, respectively, which is significantly superior to other methods, indicating that our model has excellent performance for the rhythm classification task of ECG. Similarly, in the form task, our method has a SEN of 58.81% and an AUC of 88.22%. Compared with other methods, our model also performs well in the task of form detection. On the comprehensive tasks of super-diag., sub-diag., diag., and all, our approach achieves the best results on both SEN and AUC. It achieves 80.17%, 73.28%, 68.54%, 72.96% (SEN) and 92.90%, 92.88%, 93.78%, 93.14% (AUC), respectively, showing comprehensive and stable performance. It is worth noting that in the comparative experiment, our method achieved higher sensitivity and AUC on multiple tasks compared with other models, indicating that our model has better processing ability for different ECG tasks.

**TABLE 2:** Comparison of various methods with our proposed method across multiple tasks of the PTB-XL. Bold values indicate the best results.

Method	Rhythm		Form		Super-diag		Sub-diag		Diag		All	
	SEN	AUC	SEN	AUC	SEN	AUC	SEN	AUC	SEN	AUC	SEN	AUC
MiniRocket (Dempster et al., 2021)	62.96	51.71	**60.72**	54.05	69.45	73.00	67.41	62.06	**69.31**	57.24	67.21	60.38
ViT (Dosovitskiy et al., 2020)	81.26	77.37	21.43	71.61	61.49	81.64	51.83	83.04	48.63	81.73	57.69	78.23
ATI-CNN (Yao et al., 2020)	91.17	96.69	55.58	83.54	77.77	91.81	70.34	90.80	67.17	90.83	70.23	89.51
ACNet (Chen et al., 2020)	77.82	95.90	47.65	83.12	78.14	92.33	67.45	89.69	66.27	89.51	67.81	89.54
MobileNetV3 (Howard et al., 2019)	91.39	96.22	48.25	81.80	75.40	91.29	64.06	89.25	61.34	89.53	68.81	90.12
Xresnetad101 (He et al., 2019)	91.15	94.90	51.35	82.02	75.27	91.95	67.94	89.98	64.69	91.42	68.89	90.61
resnet1d_wang (Wang et al., 2017)	89.71	95.06	52.97	86.04	75.43	92.17	68.23	90.91	66.44	92.12	69.32	91.13
fcn_wang (Wang et al., 2017)	88.37	92.61	49.42	85.43	76.42	92.02	69.90	90.15	65.63	91.97	68.88	91.24
BiLSTM (Zhang et al., 2015)	88.42	94.93	49.24	82.14	78.61	92.19	69.13	91.98	64.13	91.37	69.53	91.35
InceptionTime (Ismail Fawaz et al., 2020)	90.11	95.03	59.05	86.66	78.17	92.61	70.95	92.39	66.24	92.82	71.93	92.11
LSTM (Hochreiter and Schmidhuber, 1997)	87.19	91.27	48.82	85.14	76.21	92.44	67.36	91.66	64.28	91.72	67.97	92.74
Our	**91.68**	**97.02**	58.81	**88.22**	**80.17**	**92.90**	**73.28**	**92.88**	68.54	**93.78**	**72.96**	**93.14**

Table 3 shows the performance of our proposed method compared with other advanced methods on CPSC2018 and HFHC datasets. On the CPSC2018 dataset, our method achieved 95.88% and 84.49% on AUC and SEN, respectively, showing significant advantages compared with other methods. In particular, compared with the suboptimal method, our model improves AUC by at least 0.65% and SEN by at least 3.25%. This shows that our model has a stronger classification ability on the CPSC2018 dataset and can identify the relevant features of ECG more accurately. On the HFHC dataset, our method also achieved excellent performance, with AUC and SEN reaching 94.96% and 95.73%, respectively. Compared to other methods, our model improves at least 2.18% on AUC and 3.26% on SEN. This further validates the superiority of our model on HFHC datasets with higher sensitivity and better classification performance.

**TABLE 3:** Comparison of various methods with our proposed method across multiple tasks of the CPSC2018 dataset and the HFHC dataset. Bold values indicate the best results.

Method	CPSC				HFHC			
	AUC	SEN	$F_{1}$	Recall	AUC	SEN	$F_{1}$	Recall
ViT (Dosovitskiy et al., 2020)	82.58	47.10	64.89	69.35	61.17	35.70	66.18	68.49
MiniRocket (Dempster et al., 2021)	73.23	60.49	66.82	73.18	67.39	62.42	60.26	62.58
fcn_wang (Wang et al., 2017)	91.85	70.59	69.80	75.58	89.08	84.71	73.17	69.90
LSTM (Hochreiter and Schmidhuber, 1997)	94.81	74.93	72.41	78.84	89.82	87.31	71.58	70.22
BiLSTM (Zhang et al., 2015)	95.06	77.75	76.65	81.52	91.21	88.43	76.64	71.15
ACNet (Chen et al., 2020)	94.66	80.94	58.15	74.35	91.46	90.73	70.21	69.82
Xresnet1d101 (He et al., 2019)	95.22	82.61	79.78	81.94	90.88	90.87	79.94	74.55
InceptionTime (Ismail Fawaz et al., 2020)	94.47	79.28	76.84	82.25	92.00	90.89	82.19	72.17
resnet1d_wang (Wang et al., 2017)	94.72	76.74	75.52	80.36	92.18	91.23	85.58	79.63
MobileNetV3 (Howard et al., 2019)	95.23	81.52	75.64	79.35	92.77	91.55	88.27	78.84
ATI-CNN (Yao et al., 2020)	94.73	83.26	76.63	82.54	91.85	92.47	84.15	80.52
Ours	**95.88**	**84.49**	**82.14**	**83.60**	**94.96**	**95.73**	**90.24**	**82.57**

Table 4 presents a comparison of our proposed method with other networks on various tasks within the Chapman dataset. Our model showcases remarkable performance, achieving a notable $F_{1}$ score of 97.21%, which positions it as one of the top-performing models. Although our AUC of 99.12% is not the highest, it remains competitive among the compared methods. Specifically, when compared to the model with the highest AUC (Zhang et al., 2023), our approach demonstrates a strong performance, trailing behind by only 0.38%. In terms of recall, our method outperforms all others with a leading score of 96.84%, emphasizing its effectiveness in capturing true positive instances. The overall accuracy of 97.12% further substantiates the robustness of our proposed model in ECG classification.

**TABLE 4:** Comparison of $F_{1}$ score, macro-auc, recall, and accuracy of various methods with our proposed method across multiple tasks of the Chapman dataset. Bold values indicate the best results.

Method	Chapman			
	$F_{1}$	AUC	Recall	Accuracy
LSTM (Hochreiter and Schmidhuber, 1997)	91.79	97.53	91.82	92.58
BiLSTM (Zhang et al., 2015)	93.64	98.53	93.75	94.18
resnet1d_wang (Wang et al., 2017)	92.47	98.40	92.63	93.05
fcn_wang (Wang et al., 2017)	83.88	93.95	83.26	85.54
ViT (Dosovitskiy et al., 2020)	81.60	95.72	82.06	83.19
ACNet (Chen et al., 2020)	91.26	98.52	91.34	92.02
ATI_CNN (Yao et al., 2020)	94.76	72.87	93.80	95.21
Xresnet1d101 (He et al., 2019)	95.00	99.14	94.98	95.40
InceptionTime (Ismail Fawaz et al., 2020)	92.20	98.14	92.50	92.96
MobileNetV3 (Howard et al., 2019)	94.00	99.08	94.08	94.55
ECGNet (Lynn et al., 2019)	95.98	99.39	96.07	95.02
ECG_BNN (Murugesan et al., 2018)	92.58	98.68	92.63	93.24
MVMSNet (Yang et al., 2023)	93.30	98.26	93.39	93.80
MSEL (Zhang et al., 2023)	96.52	**99.50**	96.44	96.90
Ours	**97.21**	99.12	**96.84**	**97.12**

**FIGURE 5:**
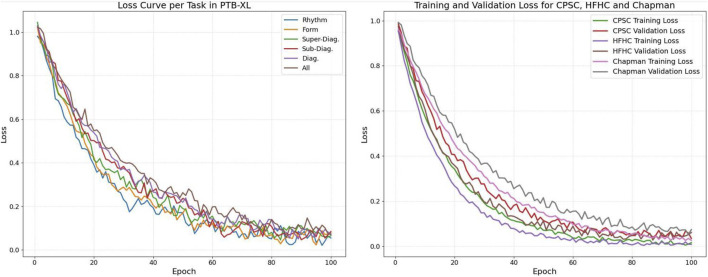
Loss of different datasets.

Figure 5 illustrates the loss curves across different datasets and tasks, providing a comprehensive view of the model’s learning dynamics. As shown in the left subfigure, the training losses for the six PTB-XL subtasks—including Rhythm, Form, Super-Diagnostic, Sub-Diagnostic, Diagnostic, and All—exhibit a consistent downward trend with moderate fluctuations, indicating stable convergence. The right subfigure presents both training and validation losses for CPSC, HFHC, and Chapman datasets. The clear and steadily decreasing validation curves, along with the narrowing gaps between training and validation loss, suggest that the model effectively avoids overfitting and generalizes well across datasets.

### 4.4 Ablation studies

In this section, we conduct ablation experiments to evaluate the effects of the time domain features embedding module *versus* the causal reasoning module. At the same time, we investigate the effects of the convolution kernel size of the time domain features embedding module on ECG classification performance.

#### 4.4.1 Impact of kernel size

Table 5 shows the performance comparison of different convolution kernel combinations in time-domain features embedding, covering both the PTB-XL dataset and the Chapman dataset. We focused on several key metrics to assess the impact of different convolution kernel combinations on feature extraction in the time domain.

**TABLE 5:** Performance of various kernel sizes in Time Domain Features Embedding on the PTB-XL dataset and the Chapman dataset. Bold values indicate the best results.

Kernel size		PTB-XL				Chapman			
U	L	$F_{1}$	AUC	Recall	mAP	$F_{1}$	AUC	Recall	Acc
40	20	**34.23**	84.02	**63.20**	74.04	93.11	96.04	92.98	94.02
40	10	32.04	**85.22**	58.25	**76.31**	**93.85**	**97.08**	**94.22**	93.14
50	20	31.88	83.12	59.45	73.21	92.83	96.86	92.90	93.90
50	10	31.92	84.82	58.88	74.29	92.55	94.45	93.80	**94.11**

On the PTB-XL dataset, the convolution kernel combinations 40/20 perform best in terms of $F_{1}$ score and recall rate, which are 34.23% and 63.20% respectively. This shows that this combination has a good balance in time domain feature extraction and can capture key features more accurately. On the Chapman dataset, the 40/10 down convolution kernel combination has the best performance, which are 93.85%, 97.08%, and 94.22%, respectively. This shows that this convolution kernel combination can achieve the best performance in time domain feature extraction.

In general, the choice of convolution kernel combination is very important for the extraction of time domain features. This reminds us that different combinations of convolution kernel may be required on different tasks and data sets to optimize the performance of feature extraction in the time domain.

#### 4.4.2 Module ablation experiment

Table 6 shows the performance comparison of the time domain embedding module (TDFE), causal inference module (CR), and their combination (TDFE + CR) under different tasks. In the rhythm task, the TDFE module achieved 91.74% and 86.39% on AUC and SEN, and the CR module achieved 91.22% and 88.42% on AUC and SEN, respectively. However, the TDFE + CR combination performed the best, with AUC and SEN reaching 97.02% and 91.68%, respectively, a significant improvement over any single module. This shows that both TDFE and CR modules play an active role in rhythm tasks, and their combination effect is better.

**TABLE 6:** Performance of Time Domain Features Embedding (TDFE) and Causal Reasoning module (CR), as well as their combination (TDFE + CR). Bold values indicate the best results.

Task		×	TDFE	CR	TDFE + CR
rhythm	AUC	80.23	91.74	91.22	**97.02**
	SEN	80.27	86.39	88.42	**91.68**
form	AUC	76.76	81.30	84.15	**88.22**
	SEN	37.24	44.58	49.82	**58.81**
super-diag	AUC	82.09	88.77	86.26	**92.90**
	SEN	69.88	74.86	75.28	**80.17**
sub-diag	AUC	82.84	89.94	88.68	**92.88**
	SEN	59.23	67.82	66.57	**73.28**
diag	AUC	83.32	86.72	89.01	**93.78**
	SEN	57.67	60.12	65.33	**68.54**
all	AUC	74.48	83.88	86.73	**93.14**
	SEN	62.01	67.05	69.50	**72.96**

In the form task, the TDFE module achieved 81.30% and 44.58% on AUC and SEN, and the CR module achieved 84.15% and 49.82% on AUC and SEN, respectively. The TDFE + CR combination achieved 88.22% and 58.81% on AUC and SEN, respectively, significantly outperforming a single module. This shows that both TDFE and CR modules are beneficial in form tasks, and their combination is more significant. Similar trends are observed in other tasks (super-diag., sub-diag., diag., all). Overall, the TDFE and CR modules had a positive impact across the tasks, and their combination was even more significant, providing more powerful performance for the ECG classification task. This further validates the effectiveness of comprehensive treatment of confounders by obtaining image features and temporal domain features and discovering true causal associations.

#### 4.4.3 Parameters analysis

As shown in Table 7, our model performs well on both the number of parameters and the inference time. Specifically, we had the second smallest number of participants at 0.25 million. The inference time was only 2.27 milliseconds, the second fastest. Compared to other methods, the number of parameters in our model is much lower than most other methods, only 0.25 million. This means that our model has a more concise structure, and fewer parameters help to reduce the complexity of the model, reduce the risk of overfitting, and save computing resources. Second, our model’s inference time is also excellent, at just 2.27 milliseconds. Although there are some methods with shorter inference times, our model is still at a faster level. This means that our model has high real-time and efficiency in practical applications, and can process data quickly. This indicates that our model is less complex and the cost of implementing causal reasoning is not high.

**TABLE 7:** The number of parameters and inference time for each method. Bold values indicate the best results.

Method	Parameters $\left(10^{6}\right)$	Infer time (ms)
LSTM (Hochreiter and Schmidhuber, 1997)	0.81	35.18
ACNet (Chen et al., 2020)	**0.03**	3.55
resnet1d_wang (Wang et al., 2017)	0.29	**1.70**
MobileNetV3 (Howard et al., 2019)	1.48	9.04
MVMSNet (Yang et al., 2023)	0.39	8.91
Ours	0.25	2.27

## 5 Discussions

Causal reasoning plays a pivotal role in ECG classification, particularly in scenarios where cardiac abnormalities may be influenced by infectious diseases. By leveraging causal reasoning, we gain deeper insights into the causal relationships between features in ECG signals and their connections to classification outcomes, thereby enhancing both the accuracy and interpretability of classification models.

In this study, causal reasoning helps uncover the underlying causal chains in ECG signals, revealing how different physiological events and pathological states, such as those induced by infections, interact with cardiac function. This understanding is crucial for medical professionals, as it provides a clearer perspective on the patient’s condition, aiding in the timely and precise diagnosis and treatment of heart conditions potentially linked to infectious diseases. By identifying these cause-and-effect relationships, causal reasoning allows us to design more effective feature extraction and selection methods. These methods improve the model’s sensitivity to clinically relevant information while minimizing its susceptibility to noise and confounding effects. Moreover, causal reasoning contributes to building more reliable and generalizable models by reducing overfitting and ensuring a clearer direction of causality during model training. This ultimately has a direct impact on the early detection and treatment of heart diseases, particularly those influenced by infectious agents, improving the quality of medical services.

Our study specifically addresses the removal of confounding factors in ECG classification tasks by employing backdoor adjustment techniques. This approach enhances classification performance by re-weighting attention mechanisms and refining the representation of confounding factors. Experimental results demonstrate that the proposed causal attention module effectively mitigates the impact of confounders, significantly improving classification accuracy. By dynamically adjusting attention weights and optimizing the confounding dictionary representation, our method directs the model’s focus toward features most relevant to ECG classification, particularly in infection-related cases. This confounding removal strategy not only boosts model performance but also improves its interpretability and reliability, providing a valuable tool for clinical applications. Such advancements are essential in aiding healthcare professionals in diagnosing and managing cardiac conditions, especially those influenced by infectious diseases, with greater precision and confidence.

## 6 Conclusion

In this study, we propose an innovative ECG signal classification network that integrates temporal feature embedding and attention-based causal inference, addressing the challenges of multi-label ECG classification in medical scenarios, including infectious diseases. The network employs a convolutional architecture and dual temporal feature embedding modules to efficiently learn temporal patterns in ECG signals, enabling accurate classification in complex clinical settings. To enhance causal reasoning, we introduce confounding dictionaries and attention mechanisms, allowing the network to capture high-quality ECG information while mitigating the effects of confounding factors. Specifically, the proposed causal reasoning framework models the causal relationships in ECG signals, identifies true causal connections, and eliminates confounding effects, which are critical for distinguishing cardiac abnormalities potentially caused by infections, such as myocarditis or sepsis-induced cardiac dysfunction. Experimental results demonstrate that our network achieves competitive performance, highlighting its potential in diagnosing infectious disease-related cardiac complications.

## Data Availability

The original contributions presented in the study are included in the article/supplementary material, further inquiries can be directed to the corresponding author.
